# Experimental evidence for direct insulator-quantum Hall transition in multi-layer graphene

**DOI:** 10.1186/1556-276X-8-214

**Published:** 2013-05-06

**Authors:** Chiashain Chuang, Li-Hung Lin, Nobuyuki Aoki, Takahiro Ouchi, Akram M Mahjoub, Tak-Pong Woo, Jonathan P Bird, Yuichi Ochiai, Shun-Tsung Lo, Chi-Te Liang

**Affiliations:** 1 Department of Physics, National Taiwan University, Taipei 106, Taiwan; 2 Graduate School of Advanced Integration Science, Chiba University, Chiba 263-8522, Japan; 3 Department of Electrophysics, National Chiayi University, Chiayi 600, Taiwan; 4 Department of Electrical Engineering, University at Buffalo, The State University of New York, Buffalo, NY 14206-1500, USA; 5 Graduate Institute of Applied Physics, National Taiwan University, Taipei 106, Taiwan

**Keywords:** Insulator-quantum Hall transition, Graphene flake, Multi-layer graphene

## Abstract

We have performed magnetotransport measurements on a multi-layer graphene flake. At the crossing magnetic field *B*_c_, an approximately temperature-independent point in the measured longitudinal resistivity *ρ*_*xx*_, which is ascribed to the direct insulator-quantum Hall (I-QH) transition, is observed. By analyzing the amplitudes of the magnetoresistivity oscillations, we are able to measure the quantum mobility *μ*_q_ of our device. It is found that at the direct I-QH transition, *μ*_q_*B*_c_ ≈ 0.37 which is considerably smaller than 1. In contrast, at *B*_c_, *ρ*_*xx*_ is close to the Hall resistivity *ρ*_*xy*_, i.e., the classical mobility *μB*_c_ is *≈* 1. Therefore, our results suggest that different mobilities need to be introduced for the direct I-QH transition observed in multi-layered graphene. Combined with existing experimental results obtained in various material systems, our data obtained on graphene suggest that the direct I-QH transition is a universal effect in 2D.

## Background

Graphene, which is an ideal two-dimensional system [1], has attracted a great deal of worldwide interest. Interesting effects such as Berry's phase [2,3] and fractional quantum Hall effect [4-6] have been observed in mechanically exfoliated graphene flakes [1]. In addition to its extraordinary electrical properties, graphene possesses great mechanical [7], optical [8], and thermal [9] characteristics.

The insulator-quantum Hall (I-QH) transition [10-13] is a fascinating physical phenomenon in the field of two-dimensional (2D) physics. In particular, a direct transition from an insulator to a high Landau-level filling factor *ν* > 2 QH state which is normally dubbed as the direct I-QH transition continues to attract interest [14]. The direct I-QH transition has been observed in various systems such as SiGe hole gas [14], GaAs multiple quantum well devices [15], GaAs two-dimensional electron gases (2DEGs) containing InAs quantum dots [16-18], a delta-doped GaAs quantum well with additional modulation doping [19,20], GaN-based 2DEGs grown on sapphire [21] and on Si [22], InAs-based 2DEGs [23], and even some conventional GaAs-based 2DEGs [24], suggesting that it is a universal effect. Although some quantum phase transitions, such as plateau-plateau transitions [25] and metal-to-insulator transitions [26-29], have been observed in single-layer graphene and insulating behavior has been observed in disordered graphene such as hydrogenated graphene [30-33], graphene exposed to ozone [34], reduced graphene oxide [35], and fluorinated graphene [36,37], the direct I-QH transition has not been observed in a graphene-based system. It is worth mentioning that the Anderson localization effect, an important signature of strong localization which may be affected by a magnetic field applied perpendicular to the graphene plane, was observed in a double-layer graphene heterostructure [38], but not in single-layer pristine graphene. Moreover, the disorder of single graphene is normally lower than those of multi-layer graphene devices. Since one needs sufficient disorder in order to see the I-QH transition [11], multi-layer graphene seems to be a suitable choice for studying such a transition in a pristine graphene-based system. Besides, the top and bottom layers may isolate the environmental impurities [39-42], making multi-layer graphene a stable and suitable system for observing the I-QH transition.

In this paper, we report magnetotransport measurements on a multi-layer graphene flake. We observe an approximately temperature-independent point in the measured longitudinal resistivity *ρ*_*xx*_ which can be ascribed to experimental evidence for the direct I-QH transition. At the crossing field *B*_c_ in which *ρ*_*xx*_ is approximately *T*-independent, *ρ*_*xx*_ is close to *ρ*_*xy*_. In contrast, the product of the quantum mobility determined from the oscillations in *ρ*_*xx*_ and *B*_c_ is *≈* 0.37 which is considerably smaller than 1. Thus, our experimental results suggest that different mobilities need to be introduced when considering the direct I-QH transition in graphene-based devices.

## Methods

A multi-layer graphene flake, mechanically exfoliated from natural graphite, was deposited onto a 300-nm-thick SiO_2_/Si substrate. Optical microscopy was used to locate the graphene flakes, and the thickness of multi-layer graphene is 3.5 nm, checked by atomic force microscopy. Therefore, the layer number of our graphene device is around ten according to the 3.4 Å graphene inter-layer distance [1,43]. Ti/Au contacts were deposited on the multi-layer graphene flake by electron-beam lithography and lift-off process. The multi-layer graphene flake was made into a Hall bar pattern with a length-to-width ratio of 2.5 by oxygen plasma etching process [44]. Similar to the work done using disordered graphene, our graphene flakes did not undergo a post-exfoliation annealing treatment [45,46]. The magnetoresistivity of the graphene device was measured using standard AC lock-in technique at 19 Hz with a constant current *I* = 20 nA in a He^3^ cryostat equipped with a superconducting magnet.

## Results and discussion

**Figure 1:**
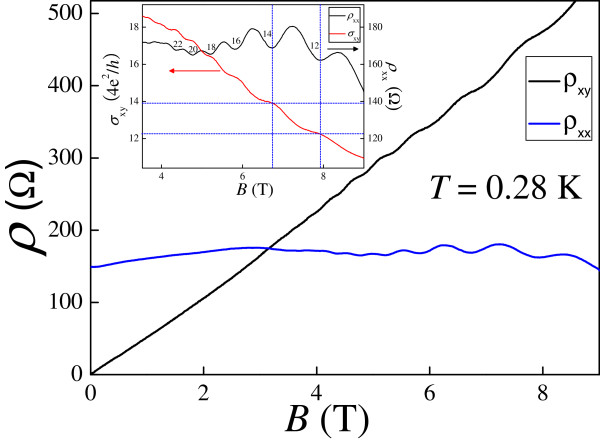
**Longitudinal and Hall resistivity *ρ***_***xx***_**(*B*) and *ρ***_***xy***_**(*B*) at *T *= 0.28 K.** The inset shows the converted *ρ*_*xy*_ (in units of 4*e*^2^/*h* ) and *ρ*_*xx*_ as a function of *B*.

Figure 1 shows the curves of longitudinal and Hall resistivity *ρ*_*xx*_(*B*) and *ρ*_*xy*_(*B*) at *T* = 0.28 K. Features of magnetoresistivity oscillations accompanied by quantum Hall steps are observed at high fields. In order to further study these results, we analyze the positions of the extrema of the magnetoresistivity oscillations in *B* as well as the heights of the QH steps. Although the steps in the converted Hall conductivity *ρ*_*xy*_ are not well quantized in units of 4*e*^2^/*h*, they allow us to determine the Landau-level filling factor as indicated in the inset of Figure 1. The carrier density of our device is calculated to be 9.4 × 10^16^ m^−2^ following the procedure described in [47,48].

**Figure 2:**
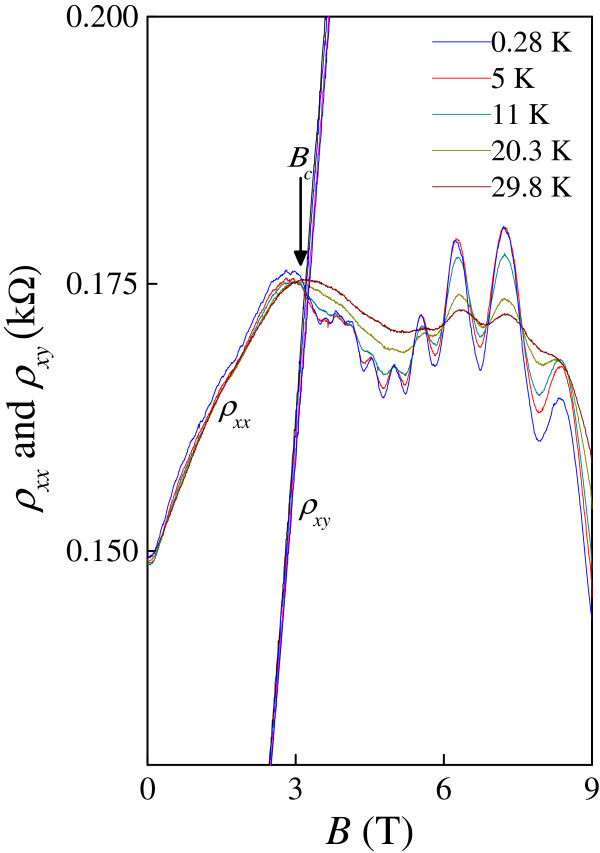
**Longitudinal and Hall resistivity *ρ***_***xx***_**(*B*) and *ρ***_***xy***_**(*B*) at various temperatures *T*.** An approximately *T*-independent point in *ρ*_*xx*_ is indicated by a crossing field *B*_c_.

We now turn to our main experimental finding. Figure 2 shows the curves of *ρ*_*xx*_(*B*) and ρ_*xy*_(*B*) as a function of magnetic field at various temperatures *T*. An approximately *T*-independent point in the measured *ρ*_*xx*_ at *B*_c_ = 3.1 T is observed. In the vicinity of *B*_c_, for *B* <*B*_c_, the sample behaves as a weak insulator in the sense that *ρ*_*xx*_ decreases with increasing *T*. For *B* >*B*_c_, *ρ*_*xx*_ increases with increasing *T*, characteristic of a quantum Hall state. At *B*_c_, the corresponding Landau-level filling factor is about 125 which is much bigger than 1. Therefore, we have observed evidence for a direct insulator-quantum Hall transition in our multi-layer graphene. The crossing points for *B* > 5.43 T can be ascribed to approximately *T*-independent points near half filling factors in the conventional Shubnikov-de Haas (SdH) model [17].

**Figure 3:**
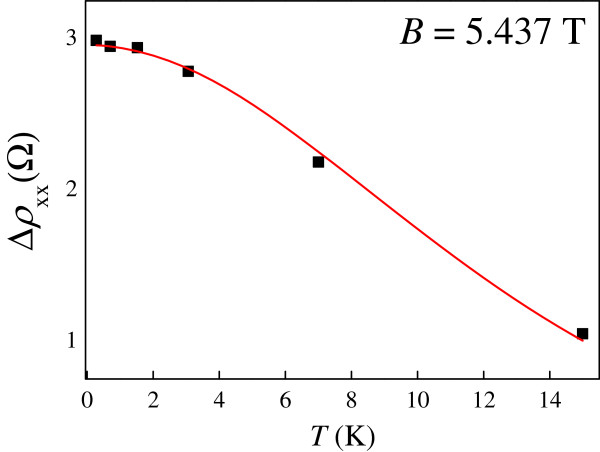
**Amplitudes of the observed oscillations Δ*ρ***_***xx***_**at *B *= 5.437 T at different temperatures.** The curve corresponds to the best fit to Equation 2.

By analyzing the amplitudes of the observed SdH oscillations at various magnetic fields and temperatures, we are able to determine the effective mass m^*^ of our device which is an important physical quantity. The amplitudes of the SdH oscillations *ρ*_*xx*_ is given by [49]:

(1) $$\Delta \rho_{\mathrm{xx}}\left(B;T\right)=4\rho_{0}\exp\left[\frac{-\pi }{\mu_{q}B}\right]D\left(B,T\right)$$

where $D\left(B,T\right)=\frac{4\pi^{3}k_{B}m^{*}T}{\mathrm{heB}}/\sinh\frac{4\pi^{3}k_{B}m^{*}T}{\mathrm{heB}}$, *ρ*_0_, *k*_B_, *h*, and *e* are a constant, the Boltzmann constant, Plank's constant, and electron charge, respectively. When $\frac{4\pi^{3}k_{B}m^{*}T}{\mathrm{heB}}>1$, we have

(2) $$\ln\frac{\Delta \rho_{\mathrm{xx}}\left(B,T\right)}{T}=C_{1}-\frac{4\pi^{3}k_{B}m^{*}T}{\mathrm{heB}}$$

where *C*_1_ is a constant. Figure 3 shows the amplitudes of the SdH oscillations at a fixed magnetic field of 5.437 T. We can see that the experimental data can be well fitted to Equation 2. The measured effective mass ranges from 0.06*m*_0_ to 0.07*m*_0_ where *m*_0_ is the rest mass of an electron. Interestingly, the measured effective mass is quite close to that in GaAs (0.067*m*_0_).

**Figure 4:**
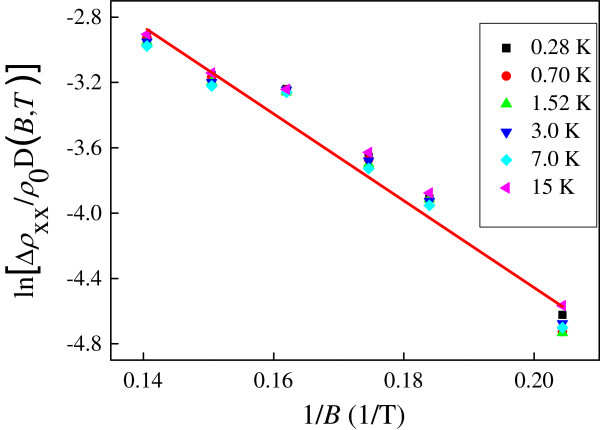
$\text{ln}\;[\frac{\Delta \rho_{\text{xx}}}{\rho_{0}D\left(B,T\right)}]$**as a function of the inverse of the magnetic field 1/*B*.** The solid line corresponds to the best fit to Equation 1.

In our system, for the direct I-QH transition near the crossing field, *ρ*_*xx*_ is close to *ρ*_*xy*_. In this case, the classical Drude mobility is approximately the inverse of the crossing field 1/*B*_c_. Therefore, the onset of Landau quantization is expected to take place near *B*_c_[50]. However, it is noted that Landau quantization should be linked with the quantum mobility, not the classical Drude mobility [19]. In order to further study the observed I-QH transition, we analyze the amplitudes of the magnetoresistivity oscillations versus the inverse of *B* at various temperatures. As shown in Figure 4, there is a good linear fit to Equation 1 which allows us to estimate the quantum mobility to be around 0.12 m^2^/V/s. Therefore, near *μ*_q_*B*_c_*≈* 0.37 which is considerably smaller than 1. Our results obtained on multi-layered graphene are consistent with those obtained in GaAs-based weakly disordered systems [19,21].

It has been shown that the elementary neutral excitations in graphene in a high magnetic field are different from those of a standard 2D system [51]. In this case, the particular Landau-level quantization in graphene yields linear magnetoplasmon modes. Moreover, instability of magnetoplasmons can be observed in layered graphene structures [52]. Therefore, in order to fully understand the observed I-QH transition in our multi-layer graphene sample, magnetoplasmon modes as well as collective phenomena may need to be considered. The spin effect should not be important in our system [53]. At present, it is unclear whether intra- and/or inter-graphene layer interactions play an important role in our system. Nevertheless, the fact that the low-field Hall resistivity is nominally *T*-independent suggests that Coulomb interactions do not seem to be dominant in our system.

## Conclusion

In conclusion, we have presented magnetoresistivity measurements on a multi-layered graphene flake. An approximately temperature-independent point in *ρ*_*xx*_ is ascribed to the direct I-QH transition. Near the crossing field *B*_c_, *ρ*_*xx*_ is close to *ρ*_*xy*_, indicating that at *B*_c_, the classical mobility is close to 1/*B*_c_ such that *B*_c_ is close to 1. On the other hand, *μ*_q_*B*_c_*≈* 0.37 which is much smaller than 1. Therefore, different mobilities must be considered for the direct I-QH transition. Together with existing experimental results obtained on various material systems, our new results obtained in a graphene-based system strongly suggest that the direct I-QH transition is a universal effect in 2D.

## Abbreviations

2D: Two-dimensional; 2DEGs: Two-dimensional electron gases; I-QH: Insulator-quantum Hall; SdH: Shubnikov-de Haas.

## Competing interests

The authors declare that they have no competing interests.

## Authors’ contributions

CC and LHL performed the experiments. CC, TO, and AMM fabricated the device. NA, YO, and JPB coordinated the project. TPW and STL provided key interpretation of the data. CC and CTL drafted the paper. All the authors read and agree the final version of the paper.
